# Supplementary material for: Investigation of In Vitro Susceptibility and Resistance Mechanisms in Skin Pathogens: Perspectives for Fluoroquinolone Therapy in Canine Pyoderma
Source: Antibiotics (Basel). 2022 Sep 6;11(9):1204. doi: 10.3390/antibiotics11091204 (PMC9494949; doi:10.3390/antibiotics11091204)
Supplement: Supplementary file 1 [file antibiotics-11-01204-s001.zip › antibiotics-1892857-supplementary.pdf]

Supplementary information file

Table S1. Quality control growth ranges. *ATCC = American Type Culture Collection

Bacterial type	Quality control	Fluoroquinolone	Range (µg/mL)	Source
S. aureus S. pseudintermedius	*S. aureus* ATCC 29213	Enrofloxacin	0.03-0.12	CLSI VET01A4 (2013)
		Marbofloxacin	0.12-0.5	CLSI VET01A4 (2013)
		Pradofloxacin	0.03-0.12	CLSI VET01A4 (2013)
		Ciprofloxacin	0.125-0.5	EUCAST 2022
		Moxifloxacin	0.016-0.125	EUCAST 2022
E. coli	*E. coli* ATCC 25922	Enrofloxacin	0.008-0.03	CLSI VET01A4 (2013)
		Marbofloxacin	0.008-0.03	CLSI VET01A4 (2013)
		Pradofloxacin	0.008-0.03	CLSI VET01A4 (2013)
		Ciprofloxacin	0.004-0.016	EUCAST 2022
		Moxifloxacin	0.008-0.06	EUCAST 2022

Table S2. Fluoroquinolones (veterinary and human) clinical breakpoints (CBP)

Bacterial type	Drug	Susceptibility CBP (µg/mL)	Resistance CBP (µg/mL)	Source of CBP	ECOFF (µg/mL)
S. aureus	ENR	<4	≥4	CLSI VET01S ED5:2020	
	MAR	<4	≥4	CLSI VET01S ED5:2020	
	PRA	<2	≥2	CLSI VET01S ED5:2020	
	CIP	<4	≥4	CLSI M100- D32:2022	1 (EUCAST 2022)
	MOX	<2	≥2	CLSI M100- D32:2022	0.25 (EUCAST 2022)
S. pseudintermedius	ENR	<4	≥4	CLSI VET01S ED5:2020	0.5 (EUCAST 2022)
	MAR	<4	≥4	CLSI VET01S ED5:2020	
	PRA	<2	≥2	CLSI VET01S ED5:2020	
	CIP	<4	≥4	CLSI M100- D32:2022	
	MOX	<2	≥2	CLSI M100- D32:2022	
E. coli	ENR	<4	≥4	CLSI VET01S ED5:2020	0.125 (EUCAST 2022)
	MAR	<4	≥4	CLSI VET01S ED5:2020	
	PRA	<2	≥2	CLSI VET01S ED5:2020	
	CIP	<1	≥1	CLSI M100- D32:2022	0.06 (EUCAST 2022)
	MOX	≤ 0.25	>0.25	EUCAST 2022	0.25 (EUCAST 2022)

Figure S1. MBC distributions in *S. aureus* (first row), *S. pseudintermedius* (second row) and *E. coli* (third row) of 5 fluoroquinolones. Blue dotted lines indicate the clinical breakpoint, the highest MIC value considered susceptible, and the epidermiological cut-off (ECOFF), the MIC value that separates wild-type from non wild-type bacteria.

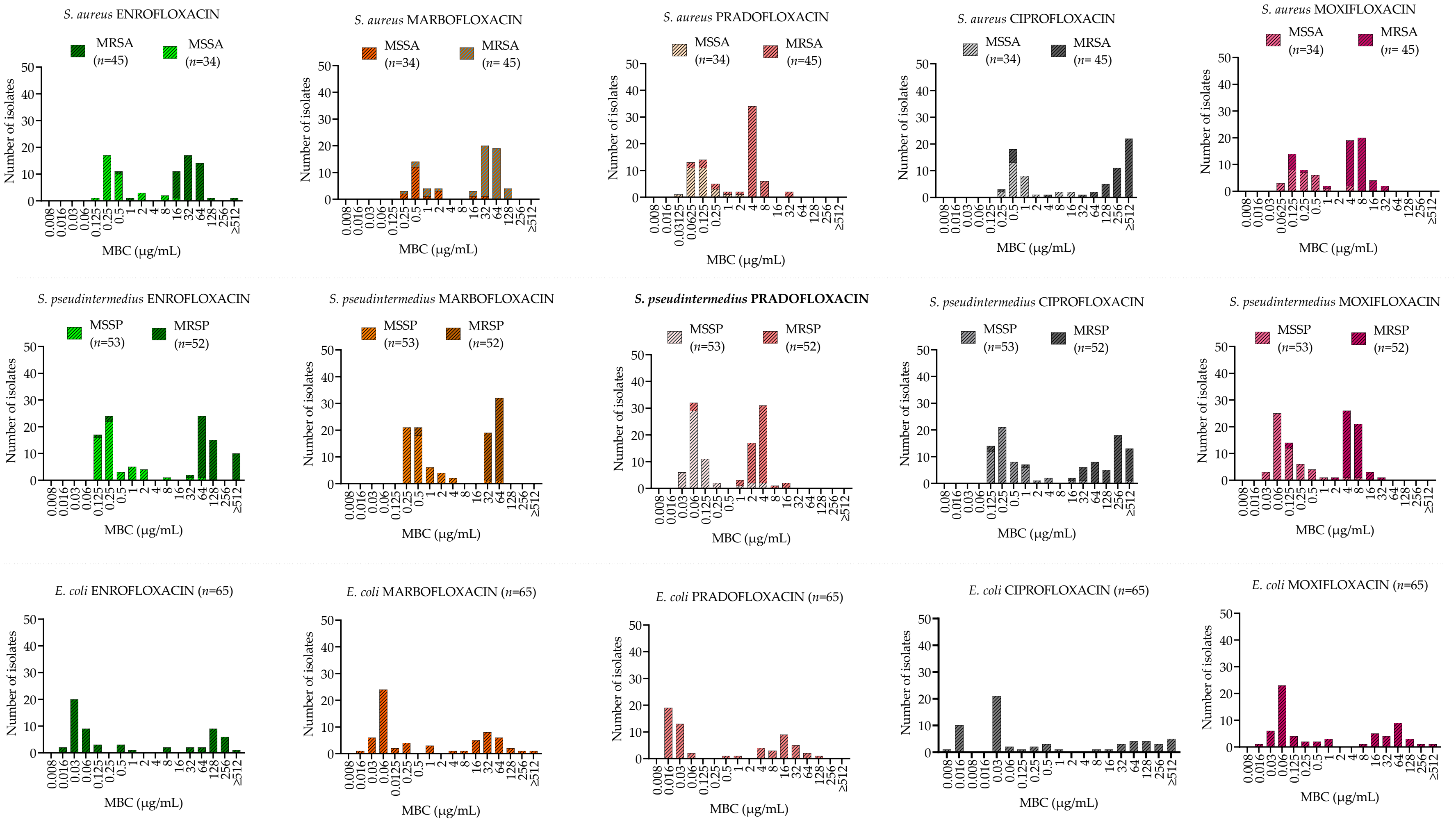

Table S3. Statistical comparison between MICs of veterinary and human FQs in *S. aureus*, *S. pseudintermedius* and *E. coli*. Anderson Darling test compared the areas of the distributions between MS, MR staphylococci and *E. coli* among the FQs. Friedman's test compared the medians (MIC_{50}) of the aforementioned distributions. Dunn's post hoc analysis was carried out for pairwise comparison between FQs.

**Bacterial species**	**Anderson Darling test + Friedman's test**	**Dunn's post hoc analysis (Pairwise comparison)**					
MSSA (n=34)<br>MRSA (n=45)	MSSA ***<br>MRSA ***		ENR	MAR	PRA	CIP	MOX
		ENR	-	MSSA *<br>MRSA (NS)	MSSA ***<br>MRSA ***	MSSA *<br>MRSA ***	MSSA (NS)<br>MRSA **
		MAR	-	-	MSSA ***<br>MRSA ***	MSSA (NS)<br>MRSA *	MSSA ***<br>MRSA ***
		PRA	-	-	-	MSSA ***<br>MRSA ***	MSSA (NS)<br>MRSA (NS)
		CIP	-	-	-	-	MSSA ***<br>MRSA ***
		MOX	-	-	-	-	-
MSSP (n=53)<br>MRSP (n=52)	MSSP ***<br>MRSP ***		ENR	MAR	PRA	CIP	MOX
		ENR	-	MSSP ***<br>MRSP (NS)	MSSP ***<br>MRSP ***	MSSP (NS)<br>MRSP (NS)	MSSP ***<br>MRSP ***
		MAR	-	-	MSSP ***<br>MRSP ***	MSSP (NS)<br>MRSP (NS)	MSSP ***<br>MRSP ***
		PRA	-	-	-	MSSP ***<br>MRSP ***	MSSP (NS)<br>MRSP (NS)
		CIP	-	-	-	-	MSSP ***<br>MRSP ***
		MOX	-	-	-	-	-
E. coli (n=65)	***		ENR	MAR	PRA	CIP	MOX
		ENR	-	(NS)	***	(NS)	(NS)
		MAR	-	-	***	(NS)	(NS)
		PRA	-	-	-	***	***
		CIP	-	-	-	-	(NS)
		MOX	-	-	-	-	-

* $p < 0.05$, ** $p < 0.01$, *** $p < 0.001$, NS = not statistically significant ($p > 0.05$).

Table S4. Statistical comparison between MBCs of veterinary and human FQs in *S. aureus*, *S. pseudintermedius* and *E. coli*. Anderson Darling test compared the areas of the distribution between MS and MR staphylococci and in *E. coli* among the FQs. Friedman's test compared the medians (MBC_{50}) of the beforementioned distributions. Dunn's post hoc analysis was carried out for pairwise comparison between FQs.

**Bacterial species**	**Anderson Darling test + Friedman's test**	**Dunn's post hoc analysis (Pairwise comparison)**					
MSSA (n=34) MRSA (n=45)	MSSA *** MRSA ***		ENR	MAR	PRA	CIP	MOX
		ENR	-	MSSA * MRSA (NS)	MSSA *** MRSA ***	MSSA * MRSA ***	MSSA (NS) MRSA ***
		MAR	-	-	MSSA *** MRSA ***	MSSA (NS) MRSA ***	MSSA *** MRSA ***
		PRA	-	-	-	MSSA *** MRSA ***	MSSA (NS) MRSA (NS)
		CIP	-	-	-	-	MSSA *** MRSA ***
		MOX	-	-	-	-	-
MSSP (n=53) MRSP (n=52)	MSSP *** MRSP ***		ENR	MAR	PRA	CIP	MOX
		ENR	-	MSSP * MRSP *	MSSP *** MRSP ***	MSSP (NS) MRSP (NS)	MSSP *** MRSP ***
		MAR	-	-	MSSP *** MRSP ***	MSSP (NS) MRSP **	MSSP *** MRSP ***
		PRA	-	-	-	MSSP *** MRSP ***	MSSP (NS) MRSP (NS)
		CIP	-	-	-	-	MSSP *** MRSP ***
		MOX	-	-	-	-	-
E. coli (n=65)	***		ENR	MAR	PRA	CIP	MOX
		ENR	-	(NS)	***	(NS)	(NS)
		MAR	-	-	***	*	(NS)
		PRA	-	-	-	***	***
		CIP	-	-	-	-	*
		MOX	-	-	-	-	-

* p <0.05, ** p <0.01, *** p<0.001, NS = not statistically significant (p>0.05)
